# Sonoclot’s usefulness in prediction of cardiopulmonary arrest prognosis: A proof of concept study

**DOI:** 10.1515/med-2022-0447

**Published:** 2022-03-02

**Authors:** Yumi Ichikawa, Kei Kawano, Mizuki Mori, Ayumi Numazaki, Yuto Aramaki, Kazunori Fukushima, Yuta Isshiki, Yusuke Sawada, Jun Nakajima, Kiyohiro Oshima

**Affiliations:** Department of Emergency Medicine, Gunma University Graduate School of Medicine, 3-19-15 Showa-machi, Maebashi, 371-8511, Gunma, Japan; Emergency Medical Center, Gunma University Hospital, 3-19-15 Showa-machi, Maebashi, 371-8511, Gunma, Japan

**Keywords:** viscoelastic blood coagulation, clot rate, out-of-hospital cardiopulmonary arrest, fibrin degradation products, D-dimer

## Abstract

The aim of the present study was to evaluate the usefulness of measuring whole blood coagulation with Sonoclot to predict return of spontaneous circulation (ROSC) in patients with out-of-hospital cardiopulmonary arrest (OHCA). This was a prospective, observational clinical study on patients with OHCA who were transferred to our emergency department between August 2016 and July 2018. Patients were divided into two groups: patients with return of spontaneous circulation (ROSC[+] group) and those without (ROSC[−] group). We compared the activated clotting time (ACT), clot rate (CR), and platelet function (PF) as measured with Sonoclot, and the fibrinogen degradation products (FDP) level and D-dimer level between the two groups. We analyzed 87 patients: 37 in the ROSC(+) and 50 in the ROSC(−) groups. Regarding ACT, CR, PF, FDP, and D-dimer, we used receiver operating characteristic (ROC) curves to examine how well each factor predicts ROSC. The area under the ROC curve (AUC) of CR was higher than that of the FDP and D-dimer levels. Among patients with cardiogenic cardiac arrest, the AUC of CR was higher than the AUCs of other coagulation factors. In conclusion, viscoelastic blood coagulation measurements using Sonoclot may be useful for predicting ROSC in OHCA patients.

## 1 Introduction

Cardiopulmonary resuscitation (CPR) for out-of-hospital cardiopulmonary arrest (OHCA) has been performed worldwide after guidelines for CPR were issued [1]. However, parameters that predict return of spontaneous circulation (ROSC) have not been established yet. The establishment of parameters that can predict ROSC in patients with OHCA is important for the judgment of whether and when CPR should be discontinued. In addition, these parameters must be able to be easily and quickly obtained in an emergency situation.

Szymanski et al. [2] reported that the D-dimer concentration and hemoglobin concentration obtained on admission were useful predictors of ROSC in patients with OHCA. These parameters are measured using plasma rather than whole blood, in which blood cells such as platelets, erythrocytes, and monocytes are first removed by centrifugation. However, blood cells play a pivotal role in coagulation and subsequent fibrinolysis [3]. Sonoclot is one of the point-of-care (POC) coagulation analyzers that can measure the viscoelastic changes in a whole blood sample with a small volume of blood in a relatively short period of time. Sonoclot provides accurate information on coagulation, fibrin gel formation, clot retraction (platelet function [PF]) and fibrinolysis. Using Sonoclot, most information on clot formation is available within 15 min. If details on PF are required, it may take up to 20–30 min [4]. The parameters that can be measured by Sonoclot include quantitative variables such as activated clotting time (ACT), clot rate (CR), and PF. The ACT is the time between activation of the sample and the beginning of initial fibrin formation. The CR shows the kinetics of clot development, and PF is derived from the timing and quality of clot retraction.

Few studies have evaluated the viscoelastic blood coagulation of OHCA patients [5,6], and no study has examined whether there is a relationship between the presence of a state of hypercoagulation as assessed by Sonoclot and ROSC in OHCA patients. The purpose of this study was to evaluate whether the presence of a state of hypercoagulation as measured by Sonoclot can predict ROSC in OHCA patients.

## 2 Methods

The protocol of this study was approved by the research ethics board of Gunma University Hospital (Registration number; 15–86). Informed consent for participation in this study was obtained from the patients or patients’ families. This study was a prospective, observational clinical study on patients with OHCA who were transferred to the emergency department of Gunma University Hospital between August 2016 and July 2018. Patients with advanced chronic kidney disease on hemodialysis, end-stage malignancy, bleeding tendency due to a blood disorder (e.g., leukemia, lymphoma, myeloma, idiopathic thrombocytopenic purpura, hemophilia), pregnancy, or age under 18 years were excluded. Patients with all causes of OHCA (both endogenous causes such as cardiac disease and exogenous causes such as trauma and poisoning) were included in this study. CPR was carried out by medical personnel at our hospital in conformity with the 2015 guidelines of the Japan Resuscitation Council (JRC) [7]. The JRC guideline for CPR, like other international guidelines such as those of the American Heart Association (AHA), is a CPR method developed in accordance with the CoSTR (International Consensus Conference on Cardiopulmonary Resuscitation and Emergency Cardiovascular Care Science with Treatment Recommendations) published by ILCOR (International Liaison Committee On Resuscitation) and does not differ significantly from other guidelines [7]. Patients in whom electrocardiographic monitoring on arrival at our hospital showed one among asystole, pulseless electrical activity, ventricular fibrillation, and pulseless ventricular tachycardia were diagnosed as having OHCA. Blood samples were collected from the femoral artery in all patients as soon as possible after arrival at our hospital, and blood coagulation analysis was performed with the Sonoclot analyzer (Sienco, Inc., Boulder, CO, USA) immediately after obtaining the blood sample.

### 2.1 Analysis of the viscoelastic changes in whole blood sample with the Sonoclot analyzer

To use the Sonoclot analyzer, a disposable glass bead cuvette is set in the Sonoclot apparatus, 360 µL of a whole blood sample is added to the cuvette within 2 min of blood sample collection, and then a vertically oscillating plastic probe is placed in the cuvette. The cuvette contained reagents for determining CR, PF, and ACT. Information on the entire hemostasis process is presented in quantitative values and a line graph, which are known as the Sonoclot signature. The data are obtained from the Sonoclot Signature viewer (Sienco, Inc., Boulder, CO, USA) [8,9,10]. The CR, which is the rate of formation of a fibrin gel from the fibrinogen in unit time, has a normal range of 11–35/min (analysis with glass beads) [11]. Hypercoagulability elevates the CR, and hypocoagulability lowers it. The nominal range of values for PF is from 0 (where “0” represents no PF, which indicates no clot retraction and is shown as a flat Sonoclot signature after fibrin formation) to 5 (where “5” represents strong PF, which indicates that clot retraction occurs sooner and is shown as a very strong signal with clearly defined, sharp peaks in the Sonoclot signature after fibrin formation) [12]. The ACT, which is the length of time between activation of the sample and the beginning of initial fibrin formation, is also measured.

The levels of fibrinogen degradation products (FDP) and D-dimer were measured by the immunoturbidimetric method using CS-2000i (Sysmex Corporation, Kobe, Japan) in the department of clinical laboratory of our hospital using a portion of the same blood sample as that used in the Sonoclot analyzer. The complete blood count was performed and the troponin I level was determined in the department of clinical laboratory of our hospital using a portion of the same blood sample as that used in the Sonoclot analyzer.

Patients in whom electrocardiographic monitoring showed one among asystole, pulseless electrical activity, ventricular fibrillation, and pulseless ventricular tachycardia were diagnosed as having cardiopulmonary arrest. Successful resuscitation was defined as detection of a spontaneous pulse at the carotid artery, femoral artery, or radial artery without CPR, followed by maintenance of a systolic pressure of ≧80 mmHg for 1 h with or without administration of vasoconstrictive agents. We defined this situation as “ROSC(+)”. Any condition other than ROSC(+) described above was defined as “ROSC(−)”. Patients were divided into two groups: the ROSC(+) group and the ROSC(−) group. We compared the ACT, CR, and PF measured by Sonoclot between the two groups. In addition, these three parameters were compared with the FDP and D-dimer levels that had been measured with a portion of the same blood sample in terms of prediction of ROSC.

### 2.2 Statistical analysis

Data are shown as median or number and percentage. The chi-square test, Fisher’s exact test, or Mann–Whitney *U* test were used for comparisons between the ROSC(+) and ROSC(−) groups. Receiver operating characteristic (ROC) curves were used to evaluate the efficacy of predicting ROSC and to determine the cut-off point with the Youden index. The optimal cut-off point is the point that maximizes the sum of sensitivity and specificity employed in the Youden index approach. IBM SPSS Statistics version 26 was used for statistical analyses. Statistical significance was set at a *p* value of less than 0.05.

## 3 Results

During the study period, 87 patients with OHCA met the study criteria. Among these 87 patients, 37 patients could obtain ROSC, and the remaining 50 died without obtaining ROSC.

The characteristics and past history of the ROSC(+) and ROSC(−) groups are summarized in Tables 1 and 2, respectively. As shown in Tables 1 and 2, there were no significant differences in age, male/female ratio, rate of bystander CPR, and past history of implantable cardioverter defibrillator between the ROSC(+) and ROSC(−) groups. The rate of OHCA witnessed by citizen personnel was significantly higher in the ROSC(+) group than in the ROSC(−) group (62.2% vs 14.0% and *p* = 0.000). There was a significant difference in the initial rhythm between the two groups. There were no significant differences in length of time from call to arrival at our hospital and the causes of cardiopulmonary arrest. As shown in Table 2, the rates of previous anticoagulant therapy, previous antiplatelet therapy, and history of arrhythmia were significantly higher in the ROSC(+) group, although there were more cases in which the history of medication and history of arrhythmia were unknown in the ROSC(−) group.

**Table 1:** Characteristics of the patients with OHCA

Variables	ROSC(−)	ROSC(+)	*p*-value
	(*n* = 50)	(*n* = 37)	
Age, years	79.52 (24–102)	80.08 (54–99)	0.667
Sex, male	23 (46.0%)	14 (62.1%)	0.149
Bystander cardiopulmonary resuscitation	22 (44.0%)	21 (56.8%)	0.889
OHCA witnessed by citizen personnel	7 (14.0%)	23 (62.2%)	0.000
OHCA not witnessed by citizen personnel	33 (66.0%)	14 (37.8%)	
Initial rhythm			0.007
VF	3 (6.0%)	3 (8.1%)	
Pulseless VT	0 (0%)	1 (2.7%)	
PEA	7 (14.0%)	16 (43.2%)	
Asystole	40 (80.0%)	17 (45.9%)	
Call to arrival time, min	27.65 (10–165)	26.35 (10–53)	0.470
Etiology			0.087
Cardiac	29 (58.0%)	14 (37.8%)	
Aortic	5 (10.0%)	2 (5.4%)	
Stroke	2 (4.0%)	1 (2.7%)	
Respiratory	6 (12.0%)	2 (5.4%)	
Asphyxia	4 (8.0%)	10 (27.0%)	
Hemorrhagic shock	2 (4.0%)	2 (5.4%)	
Trauma	0 (0%)	2 (5.4%)	
Other	2 (4.0%)	4 (10.8%)	

OHCA, out-of-hospital cardiac arrest; VF, ventricular fibrillation; VT, ventricular tachycardia; PEA, pulseless electrical activity.

**Table 2:** History of the patients with OHCA

Variables	ROSC(−)	ROSC(+)	*p*-value
	(*n* = 50)	(*n* = 37)	
Anticoagulant	1 (2%)	5 (13.5%)	0.001
Warfarin	0 (0%)	2 (5.4%)	
Direct oral anticoagulants	1 (2%)	3 (8.1%)	
Antiplatelet	1 (2%)	4 (10.8%)	0.006
Aspirin	0 (0%)	3 (8.1%)	
Clopidogrel sulfate and Cilostazol	1 (2%)	1 (2.7%)	
History of medication unknown	16 (32%)	2 (5.4%)	
History of arrhythmia			0.006
Premature ventricular contraction	0 (0%)	1 (2.7%)	
Atrial fibrillation	1 (2%)	1 (2%)	
Atrial flutter	1 (2%)	0 (0%)	
Complete atrioventricular block and sinus node syndrome	0 (0%)	2 (5.4%)	
History of arrhythmia unknown	17 (34%)	2 (5.4%)	
Implantable cardiac device	2 (4%)	3 (8.1%)	0.107
Cardiac pacemaker	2 (4%)	1 (2.7%)	
Implantable cardioverter defibrillator	0 (0%)	2 (5.4%)	

Comparisons of ACT, CR, PF, FDP, D-dimer, hematocrit, hemoglobin, white blood cell count, platelet count, and troponin I level between the ROSC(+) and ROSC(−) groups are shown in Table 3. The ACT was significantly shorter and the FDP, D-dimer, and troponin I levels were significantly lower in the ROSC(+) group than in the ROSC(−) group. On the other hand, the CR and PF were significantly higher in the ROSC(+) group than in the ROSC(−) group. The platelet count was also significantly higher in the ROSC(+) group. There were no differences in the hematocrit, hemoglobin level, and white blood cell count between the ROSC(−) and ROSC(+) groups.

**Table 3:** Comparisons of ACT, CR, PF, FDP levels, D-dimer levels, complete blood count, and troponin I levels between the ROSC(+) and ROSC(−) groups

	ROSC(−)	ROSC(+)	*p*-value*
ACT (s)	142.5 (147.67–234.54)	117.0 (97.42–139.69)	0.010
CR (clot signal/min)	18.0 (15.07–23.57)	33.0 (28.33–39.24)	0.000
PF	0.75 (0.30–1.70)	2.00 (1.35–2.33)	0.003
FDP (µg/mL)	272.9 (272.1–289.5)	36.8 (38.19–161.41)	0.000
D-dimer (µg/mL)	56.8 (85.50–244.61)	10.1 (6.53–52.00)	0.000
Hematocrit (%)	37.9 (33.45–38.67)	34.5 (32.50–37.34)	0.370
Hemoglobin (g/dL)	11.5 (10.31–12.0)	10.9 (10.31–11.92)	0.770
White blood cells (×10^3^/µL)	9.4 (8.47–12.50)	11.3 (9.47–17.3)	0.169
Platelet count (×10^3^/µL)	117.5 (109.5–160.4)	161 (145.2–204.7)	0.007
Troponin I (ng/mL)	0.37 (0.64–4.05)	0.1 (0.12–5.53)	0.015

ACT, CR, and PF were measured by the Sonoclot analyzer. ACT, activated clotting time; CR, clot rate; PF, platelet function; FDP, fibrin degradation products. Results are shown as median (25th–75th percentile). *Mann–Whitney *U* test.

**Figure 1:**
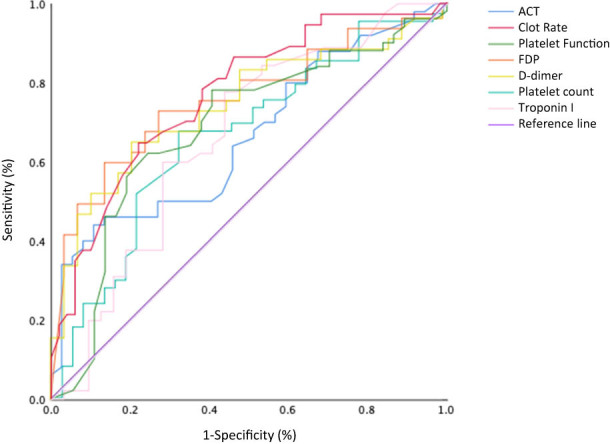
The ROC curves of ACT, CR, PF, FDP level, D-dimer level, platelet count, and troponin I level for ROSC in patients with OHCA. The ACT, CR, and PF were measured by the Sonoclot analyzer.

The ROC curves of ACT, CR, PF, FDP, D-dimer, platelet count, and troponin I levels for prediction of ROSC are shown in Figure 1, and the areas under the ROC curve (AUCs), cut-off points, and sensitivity and specificity of ACT, CR, PF, FDP, D-dimer, platelet count, and troponin I levels for prediction of ROSC are shown in Table 4. As shown in Figure 1 and Table 4, the AUC of CR was greater than the AUCs of ACT, PF, platelet count, and troponin I level, and was nearly equivalent to the AUCs of FDP and D-dimer levels.

**Table 4:** Areas under receiver operating characteristic curves (AUC) and cut-off points to predict return of spontaneous circulation

	ACT	CR	PF	FDP	D-dimer	Platelet count	Troponin I
AUC (95%CI)	0.663 (0.550–0.777)	0.770 (0.670–0.870)	0.685 (0.576–0.802)	0.761 (0.646–0.876)	0.747 (0.629–0.865)	0.663 (0.534–0.791)	0.663 (0.547–0.780)
Cut-off point	146.5	28.5	1.0	168.35	29.3	126.5	0.12
Sensitivity (%)	89.2	64.9	75.7	86.2	79.3	68.0	77.8
Specificity (%)	44.0	78.0	62.0	60.5	65.8	67.6	56.2

ACT, activated clotting time; CR, clot rate; PF, platelet function; FDP, fibrin degradation products; 95% CI, confidence interval.

**Figure 2:**
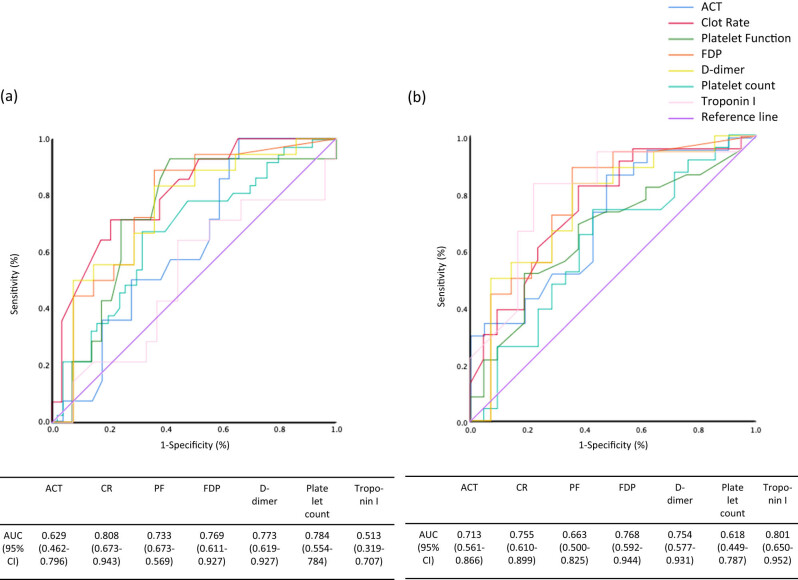
The ROC curves and AUCs of ACT, CR, PF, FDP level, D-dimer level, platelet count, and troponin I level for ROSC in patients with OHCA due to cardiac (a) or non-cardiac causes (b). The ACT, CR, and PF were measured by the Sonoclot analyzer. 95% CI, confidence interval.

In addition, we divided the 87 subjects into those who had OHCA due to cardiac (*n* = 43) or non-cardiac (*n* = 44) causes, and performed the same analyses (Figure 2). In patients who had OHCA due to cardiac causes, the AUC of CR was the largest among the AUCs of these parameters (Figure 2a). However, in patients who had OHCA due to non-cardiac causes, the AUC of CR was nearly equivalent to the AUCs of FDP and D-dimer levels, and the AUC of troponin I level was the largest among the AUCs of these parameters (Figure 2b).

## 4 Discussion

In this study, we evaluated the usefulness of analyzing whole blood coagulation with Sonoclot, which is a POC coagulation analyzer, in patients with OHCA and compared the coagulation parameters with the FDP and D-dimer levels, which had already been reported as predictors of ROSC [2]. We found that the ACT was significantly lower and the CR and PF as measured with Sonoclot were significantly higher in the ROSC(+) group than in the ROSC(−) group. The AUC of CR to predict ROSC was the largest among the AUCs of ACT, CR, and PF, and was nearly equivalent to the AUCs of FDP and D-dimer levels. In addition, the AUC of CR to predict ROSC was larger than the AUCs of FDP and D-dimer levels in patients with OHCA due to cardiac causes.

The usefulness of coagulo-fibrinolytic parameters such as FDP and D-dimer levels in predicting ROSC among patients with OHCA has already been reported. Szymanski et al. [2] evaluated the usefulness of D-dimer concentration as a predictor of mortality in patients with OHCA, and concluded that the D-dimer concentration was an independent predictor of all-cause mortality. Ono et al. [13] reported that the FDP level was most closely associated with favorable neurological outcomes in OHCA patients who successfully achieved ROSC. In addition, FDP and D-dimer levels reflect the prognosis of critically ill patients [14] including those with novel coronavirus pneumonia [15]. We have also focused on the relationship between coagulo-fibrinolytic parameters such as FDP and D-dimer levels and severely ill patients including those with OHCA and trauma, and have published our results up to the present time [16,17,18,19,20]. However, it takes time to obtain the results of these parameters, although they are needed quickly in emergency clinical situations. In addition, the D-dimer and FDP levels do not reflect true coagulation function. Whole blood contains several types of blood cells such as platelets, erythrocytes, and monocytes which play pivotal roles in coagulation and subsequent fibrinolysis [3]. POC coagulation analyzers including Sonoclot can measure the viscoelastic changes in a whole blood sample with a small volume of blood in a relatively short period of time.

Several studies have evaluated the usefulness of viscoelastic blood coagulation measurement with Sonoclot in clinical situations. Bischof et al. [10] reported that viscoelastic blood coagulation measurement with Sonoclot predicted postoperative bleeding in patients who underwent cardiac surgery. Kander et al. [21] demonstrated coagulability as measured with Sonoclot in survivors of cardiac arrest treated with hypothermia. Koami et al. [6] studied the relationship between viscoelastic blood coagulation parameters and ROSC in OHCA patients with another POC instrument, the ROTEM analyzer (ROTEM^®^ Delta, TEM International GmbH, Munich, Germany; www.rotem.de). However, no study on OHCA patients had been performed with Sonoclot.

Previous studies reported that the D-dimer level was lower in survivors of OHCA than in non-survivors and that the D-dimer level was useful for predicting ROSC [2,22]. Similar to previous reports, FDP and D-dimer levels were significantly lower in survivors than in non-survivors in the present study. During cardiac arrest, endothelial injury occurs, similar to that seen in severe sepsis, resulting in activation of the extrinsic coagulation pathway and protein C anticoagulant pathway dysfunction [2,22,23]. Increased coagulation results in increased fibrinolysis, leading to an increase in the D-dimer level.

**Figure 3:**
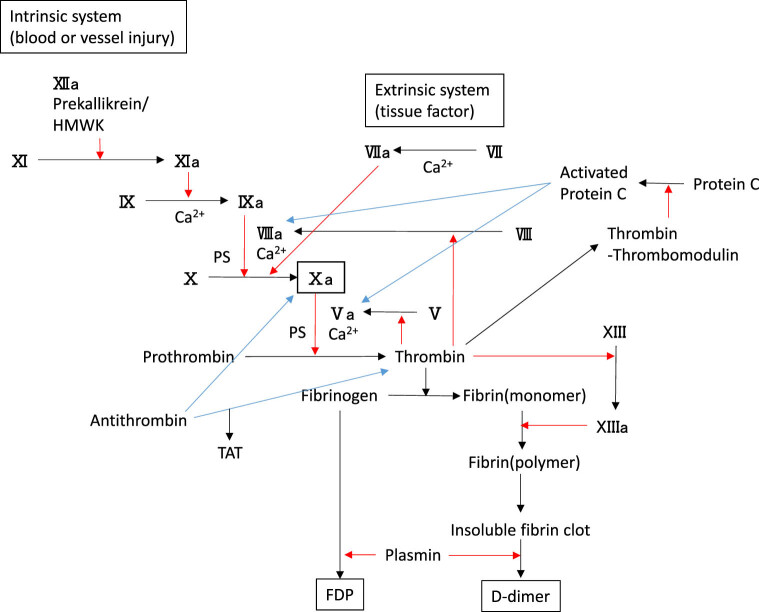
The coagulation cascade. Red arrows indicate activation, and blue arrows indicate inactivation. TAT, thrombin-antithrombin complex; HMWK, high molecular weight kininogen; PS, phosphatidylserine.

On the other hand, FDP and D-dimer are fibrinolytic markers, with D-dimer being produced as a result of both fibrin formation and fibrinolysis [24], and FDP and D-dimer levels do not directly reflect hypercoagulation. Thrombin-antithrombin complex (TAT) is a coagulation marker, but because of its short half-life of 3–15 min [25], the TAT level is not suitable for clinical assessment of coagulation. Furthermore, D-dimer and protein C are factors measured in plasma and do not reveal the entire coagulation cascade in the whole blood of patients with cardiac arrest. The coagulation cascade is shown in Figure 3 [4,24,26]. CR reflects the degree of fibrin gel formation in whole blood clotting. In other words, hypercoagulability elevates the CR, and hypocoagulability lowers it. In this study, we found that the CR was significantly higher in the ROSC(+) group than in the ROSC(−) group, indicating that the patients in the ROSC(+) group were in a state of accelerated coagulation. Among the ACT, CR, and PF measured by Sonoclot, CR was most closely related to ROSC. On the other hand, the mean CRs of the ROSC(+) and ROSC(−) groups were within the normal range and these patients were not considered to be in a state of hypercoagulation. Considering the coagulation cascade (Figure 3), hypercoagulation in the ROSC(+) group seems to be inconsistent with the lower FDP level in this group, although it was reported that the FDP level, D-dimer level, and CR are not necessarily correlated with each other [27]. In patients who are successfully resuscitated after cardiac arrest, fibrin formation is accelerated while the fibrinolytic system is also accelerated; this may indicate that hypercoagulation does not occur to the extent that the fibrinolytic system becomes excessively hyperactive. On the other hand, in the ROSC(−) group, the balance between fibrin formation and the fibrinolytic system may have been disrupted and the patients’ condition may have worsened to the point that resuscitation was not possible. A single parameter that predicts ROSC has not been established yet [2], and comprehensive judgment is required to decide whether and when to discontinue resuscitation efforts. The CR as measured with Sonoclot may help predict ROSC and determine the prognosis in patients with OHCA. Furthermore, the CR as measured with Sonoclot or other POC coagulation analyzers is clinically useful because it can be measured and evaluated earlier than the D-dimer level, the latter of which has been reported to be useful for predicting the prognosis of OHCA patients [2].

We considered the relevance of coagulability and ROSC in the etiology of OHCA, and we also examined the relationships between ACT, CR, PF, FDP, D-dimer, platelet count, or troponin I level and ROSC in patients with OHCA due to cardiac and non-cardiac causes. The AUC of troponin I level was the greatest in patients with OHCA due to non-cardiac causes; however, the AUC of troponin I level was small in patients with OHCA due to cardiac causes. In patients with OHCA due to cardiac causes, the presence of changes in troponin level in both the ROSC(−) and ROSC(+) groups may have caused the small association of troponin I level with the presence of ROSC. In the patients with OHCA due to non-cardiac causes, the troponin may have reflected myocardial damage caused by the primary disease and may have been related to the ROSC.

In patients with OHCA due to a cardiac cause, the AUC of CR was greater than the AUCs of other coagulation factors, namely, ACT, PF, FDP, and D-dimer levels. On the other hand, the AUC of CR was similar to the AUCs of other coagulation factors in patients with OHCA due to non-cardiac causes. In the present study, most of the deaths from OHCA due to non-cardiac causes were due to respiratory arrest or asphyxia, and few cases were thought to be associated with endothelial disorders such as sepsis. This result suggests that the CR may better reflect acute changes in the coagulation cascade due to endothelial injury than the D-dimer level. The details of the coagulation cascade in patients with OHCA have not been clarified and the results of this study may help to elucidate this mechanism.

### 4.1 Limitations

This study had some limitations. This was a study performed at only one institution. The number of patients was not large, and this may have been related to our finding of significant differences in patients’ characteristics between the ROSC(+) and ROSC(−) groups. There was a significant difference in the initial rhythm between the two groups. This may have been due to the fact that the percentage of OHCA cases witnessed by citizen personnel was less in the ROSC(−) group. In addition, regarding the initial rhythm in the ROSC(−) group, there was a high percentage of cases with asystole, which may have led to a longer period of time in finding that the patients were in cardiac pulmonary arrest. However, the precise length of time from the time when the patient was last seen healthy to the time when the patient was found by someone else, were not known. Also, the presence of a significant difference in the percentages of OHCA cases that were witnessed by citizen personnel between the ROSC(+) and ROSC(−) groups is a serious limitation in this study. Depending on the length of time between cardiopulmonary arrest and initiation of CPR, the results of blood coagulation may have been different.

The platelet count was significantly lower in the ROSC(−) group. There was no significant difference in causes of cardiopulmonary arrest between the ROSC(−) and ROSC(+) groups. The causes of cardiopulmonary arrest include conditions that deplete platelets such as sepsis and hemorrhagic shock, and it is possible that there was a difference in platelet counts between the two groups because the ROSC(−) group was in worse condition. Furthermore, we could not exclude patients with liver diseases because many of the OHCA patients were seen at our hospital for the first time and the Child-Pugh classification could not be determined. The small overall number of cases may also be the reason for our finding of a difference in platelet counts between the ROSC(+) and ROSC(−) groups.

Patients who suffer cardiac arrest constitute a heterogeneous population with a variety of underlying diseases and often have received multiple treatments that may interact with platelet aggregation or coagulation. In this study we did not exclude patients with a history of anticoagulation treatment, which may have affected the results. There were significant differences in the rates of history of anticoagulant or antiplatelet therapy and history of arrhythmia between the ROSC(−) and ROSC(+) groups. The reason for this finding may have been that we were not able to obtain sufficient medical history of the patients in the ROSC(−) group due to their short hospital stay. Because Sonoclot uses high frequencies, it is susceptible to mechanical shocks, which can lead to errors in the data.

## 5 Conclusion

The findings of the present proof of concept study suggest the possible use of CR as measured by Sonoclot to predict ROSC in patients with OHCA. Further studies are needed.
